# Automated brain extraction for canine magnetic resonance images

**DOI:** 10.1186/s12917-025-05003-4

**Published:** 2025-09-16

**Authors:** Gloria D. Lesta, Thomas M. Deserno, Samira Abani, Jörg Janisch, Alexej Hänsch, Merlin Laue, Stefanie Winzer, Peter J. Dickinson, Steven De Decker, Rodrigo Gutierrez-Quintana, Aleksandr Subbotin, Kseniia Bocharova, Ehren McLarty, Laura Lemke, Adriano Wang-Leandro, Franziska Spohn, Holger A. Volk, Jasmin N. Nessler

**Affiliations:** 1 DOS Software-Systeme GmbH, Wolfsburg, Germany; 2 Peter L. Reichertz Institute for Medical Informatics, TU Braunschweig and Hannover Medical School, Braunschweig, Germany; 3 Department of Small Animal Medicine and Surgery, University of Veterinary Medicine Hannover, Hanover, Germany; 4 Centre for Systems Neuroscience, University of Veterinary Medicine Hannover, Hanover, Germany; 5 Department of Surgical and Radiological Sciences, School of Veterinary Medicine, Davis, California USA; 6 Department of Clinical Science and Services, Royal Veterinary College, London, UK; 7 Small Animal Hospital, School of Biodiversity, One Health and Veterinary Medicine, University of Glasgow, Glasgow, UK

**Keywords:** Brain extraction, Skull stripping, Artificial neural networks, Magnetic resonance imaging, Neuroimaging, Brain tumour

## Abstract

**Background:**

Brain extraction is a common preprocessing step when working with intracranial medical imaging data. While several tools exist to automate the preprocessing of magnetic resonance imaging (MRI) of the human brain, none are available for canine MRIs. We present a pipeline mapping separate 2D scans to a 3D image, and a neural network for canine brain extraction.

**Methodology:**

The training dataset consisted of T1-weighted and contrast-enhanced images from 68 dogs of different breeds, all cranial conformations (mesaticephalic, dolichocephalic, brachycephalic), with several pathological conditions, taken at three institutions. Testing was performed on a similarly diverse group of 10 dogs with images from a 4th institution.

**Results:**

The model achieved excellent results in terms of Dice ($0.95\pm 0.01$) and Jaccard ($0.90\pm 0.01$) metrics and generalised well across different MRI scanners, the three aforementioned skull types, and variations in head size and breed. The pipeline was effective for a combination of one to three acquisition planes (i.e., transversal, dorsal, and sagittal). Aside from the T1 weighted imaging training datasets, the model also performed well on other MRI sequences with Jaccardian indices and median Dice scores ranging from 0.86 to 0.89 and 0.92 to 0.94, respectively.

**Conclusions:**

Our approach was robust for automated brain extraction. Variations in canine anatomy and performance degradation in multi-scanner data can largely be mitigated through normalisation and augmentation techniques. Brain extraction, as a preprocessing step, can improve the accuracy of an algorithm for abnormality classification in MRI image slices.

## Introduction

The increasing use of machine learning (ML) in medical imaging aids the automation of several research topics including disease detection, disease classification, magnetic resonance imaging (MRI) segmentation, and labelling of brain structures [1]. With that, artificial intelligence (AI) is also emerging as a research field in veterinary imaging [2, 3]. Most projects revolving around recognition or classification of brain diseases using MRI, require the brain to be extracted from the image before further preprocessing and classification is considered. This process, referred to as brain extraction, skull-stripping, or skull-scraping [4, 5], removes all non-brain tissue from the images or creates a corresponding binary mask.

While there are many tools available for brain extraction on human imaging data, it has not yet been extensively researched for subjects other than humans, non-human primates, and rodents. Human-trained models perform poorly when tested on canine MRI data [6, 7]. Therefore, several researchers manually extract dog brains to ensure a reliable analysis of the imaging data [6, 8]. To avoid manual time-consuming labelling in diseased canine MRIs, we need automatic brain extraction.

In the literature, there are only two attempts to automate brain extraction for canines. Milne et al. created four atlases for different cranial conformations [6]. They failed attempting to automatically extract canine brain using a human-tailored tool. Thus, they used manually segmented MRIs of dogs without brain diseases for the creation of their atlases. Nour Eddin et al. developed the atlas-based veterinary images brain extraction (VIBE) tool [7]. It provides models for cats and dogs, the latter using a brain atlas [9], which is generated from 30 neurologically and clinically normal non-brachycephalic dogs. The test cohort consisted of 30 dogs of different breeds and cranial conformations, with no sign of brain abnormalities.

Canine brains have a greater variability than human brains, through differences in size, shape, and cranial conformation. In addition, tissue-expanding diseases such as cancer deform the area of interest. However, structural lesions are important for disease classification. Atlas-based brain extraction in the human domain mostly consider only healthy subjects and perform less accurately when presented with MRIs with pathological conditions, such as brain tumours [10, 11]. In addition, atlas-based brain extraction often requires manual adjustment of parameters and have high processing times [12].

Therefore, the objective of this study was to develop brain extraction for diseased canines. To be beneficial for a wide range of scenarios, we aimed for an algorithm that is fully automated and applicable to dogs from various breeds, neurological diseases, and modalities. Fast computing was important to include the model as a preprocessing step in real-world applications. Fatima et al. suggested deep learning over conventional algorithms and machine learning, especially when founded on a diverse dataset [11].

In this paper, we analysed deep learning for brain extraction. As there are no publicly available datasets of labelled dog brain masks and the manual labelling is highly time-consuming and prone to intra- and inter-rater error, we used transfer learning from the human domain as initial approach and extensive data augmentation. We developed a preprocessing pipeline and segmentation model that we validated on hand-annotated scans as ground truth.

## Materials and methods

### Data acquisition

We identified suitable cases from medical records of four institutions for canine brain MRI studies between January 1, 2000 and December 31, 2022. Our database consisted of MRI and meta data from privately owned dogs with signs of encephalopathy, with written owners’ consent according to university guidelines. The MRIs yielded from different scanners and protocols, including T1-weighted (T1), T1-weighted and contrast-enhanced with Gadolinium-based contrast agent (T1ce), T2-weighted (T2), and T2-weighted-fluid-attenuated inversion recovery (T2-FLAIR). The scans were stored as individual slices for each subsectional plane or grouped as digital imaging and communications in medicine (DICOM) files. From this database, we selected a subset of T1ce scans for manual brain segmentation.

We chose this modality because it generally provides better visualisation of anatomical structures, and offers higher contrast between soft tissue and surrounding bone compared to other MRI modalities. While we defined T1ce as the primary input modality for the brain extraction model, we could register the masks to the other modalities using affine transformations, in case the dog wasn’t moved between recordings. We performed ground truth labelling following certain guidelines (Appendix A.1). As resembling experiments reached a dice score of 95% with a minimum of two to ten subjects for brain extraction on rodent and macaque MRIs [13, 14], we manually selected 10 dogs for the initial training dataset, sampling diverse characteristics representative of the population.

We evaluated the influencing factors on the accuracy of a brain extraction model for dogs on this basis, before labelling further images. For this, we used the two-dimensional (2D)-based neural brain extraction network (BEN) [14]. BEN is a framework for domain adaption of pre-trained brain extraction models. We tested the dependency on the image source (scanner hardware), dog’s head size, cranial conformation, and breed. For this, we used a homogeneous group of dogs for transfer learning and visually inspected the prediction results of unknown groups. When testing on images of the same breed and institute used for transfer learning, all test samples passed visual inspection, while only 3 of 10 image stacks of new institutes yielded adequate results. The dependency on the head size (6 out of 10) could be reduced by removing blank space around the head to 9 out of 10 acceptable predictions. 1 out of 10 dolichocephalic (D) dogs had an intolerable amount of brain tissue missing from the prediction, excluding the olfactory bulbs and adjacent frontal brain regions extending toward the nasal cavity. The remaining D and all brachycephalic (B) samples showed minor or no such segmentation issues. Predictions on images of another breed of the same size and cranial conformation did not change the quality of the results (10 out of 10).

Concerning these influencing factors, we randomly selected further images for labelling, providing a diverse dataset with several scanner sites, cranial conformations, and dog sizes. Our final study population consisted of 80 dogs (Table 1).

**Table 1:** Clinical information and groupings of the dataset

Institution	Dogs	Breeds	Head shapes	Sizes	Diagnoses
A	40	Labrador (7), German Shepherd (5), Golden Retriever (4), Poodle mix (3), ...	19 M, 6 B, 6 D, rest unknown	6 small, 9 medium, 24 big	Choroid plexus tumor (12), meningioma (7), fungal meningoencephalitis (5), ependyoma (5), carcinoma (4), fungal encephalitis (3), papilloma (2), actinomyces meningoencephalitis (1), granulomatous meningoencephalitis (GME) (1)
B	9	German Shepherd (6), Rottweiler (1), Bichon Frise (1), Labrador (1)	2 M, 6 D	1 small, 8 big	Meningioma (6), histiocytic sarcoma (1), adenocarcinoma (1), round cell neoplasm (1)
C	17	Labrador (5), Bichon Frise (4), Chihuahua (2), Pug (2), German Shepherd (1), Shetland Sheepdog (1), Shih-Tzu (1)	11 M, 3 B, 3 D	9 small, 2 medium, 7 big	meningoencephalitis of unknown origin (MUO) (14), GME (2), necrotizing meningoencephalitis (NME) (1)
D	10	Labrador Retriever (4), Boxer (3), Airedale Terrier (2), French Bulldog (1)	4 M, 4 B, 2 D	1 medium, 9 big	idiopathic epilepsy (8), meningioma (2)

Head shapes were derived by breed or manual inspection of the images. The dogs were divided into three groups of sizes by weight: small ($<5.5\textrm{kg}$), medium ($5.5\textrm{kg}$ to $25\textrm{kg}$) and big ($25\textrm{kg}$)

According to Isensee et al. [15], such a volume of data enables from-scratch training of an nnUNet, which is a three-dimensional (3D)-based model for semantic segmentation in biomedicine. It automatically adapts to a given dataset and configures a U-Net-based segmentation pipeline matching the training cases. In our approach, the training dataset consisted only of dogs with pathologies, while the final test data (institute D) included a mixture of dogs without visible brain abnormalities (e.g idiopathic epilepsy) as well as dogs with visible anomalies in the brain.

### Imaging protocol

We created the brain extraction pipeline for T1ce images of different resolutions and varying number of acquisition planes transversal (TRA), dorsal (DOR), and sagittal (SAG). The images from four institutes had different manufacturers: GE Medical Systems, Philips, and Siemens. A breakdown of the DICOM tag *DeviceSerialNumber* showed a total of ten unique devices. The in-plane pixel resolution and the slice thickness ranged from $0.18 \textrm{mm}$ to $0.86 \textrm{mm}$ and from $1.5 \textrm{mm}$ to $5 \textrm{mm}$, respectively, demonstrating the variety of our data. Furthermore, our sites stored the images at different angles and orientations (Table 2).

**Table 2:** T1ce Imaging protocol of the four institutes providing MRI data

Scanner info \ Institute	Unit	A	B	C	D
EchoTime	ms	[9.0..99.53]	[13.0..14.5]	[9.0..17.0]	[5.08..5.28]
RepetitionTime	ms	[316.66..5000.0]	[432.0..770.0]	[401.96..792.2]	[11.08..11.44]
ImagingFrequency	Hz	[63.83..63.86]	[63.6..63.9]	[63.89..63.9]	[127.72..127.76]
PixelBandwidth	hz/px	[48.83..162.73]	[160.0..160.0]	[108.55..114.47]	[133.0..135.0]
FlipAngle	°	90	90, 150	None, 90, 69	8
SliceThickness	mm	[2.0..5.0]	[3.0..4.0]	[3.0..4.5]	[1.5..1.9]
PixelSpacing	mm	[0.18..0.86]	[0.38..0.53]	[0.27..0.7]	[0.25..0.69]
Image resolution (rows x columns)	px	256^2^, 512^2^	256x224, 256^2^, 320^2^, 320x256, 320x280	160^2^, 176^2^, 224^2^, 240^2^, 256^2^, 512^2^	320^2^, 1024^2^
#slices: mean [min-max]		23 [14..102]	19 [15..24]	18 [10..23]	45 [29..72]
total #devices		4	2	3	1
Manufacturer		GE	Philips, SIEMENS	Philips	Philips

Scanner information is queried from the DICOM metadata and summarized as a list of unique values, or a range from minimum to maximum value

### Data preparation

**Fig. 1:**
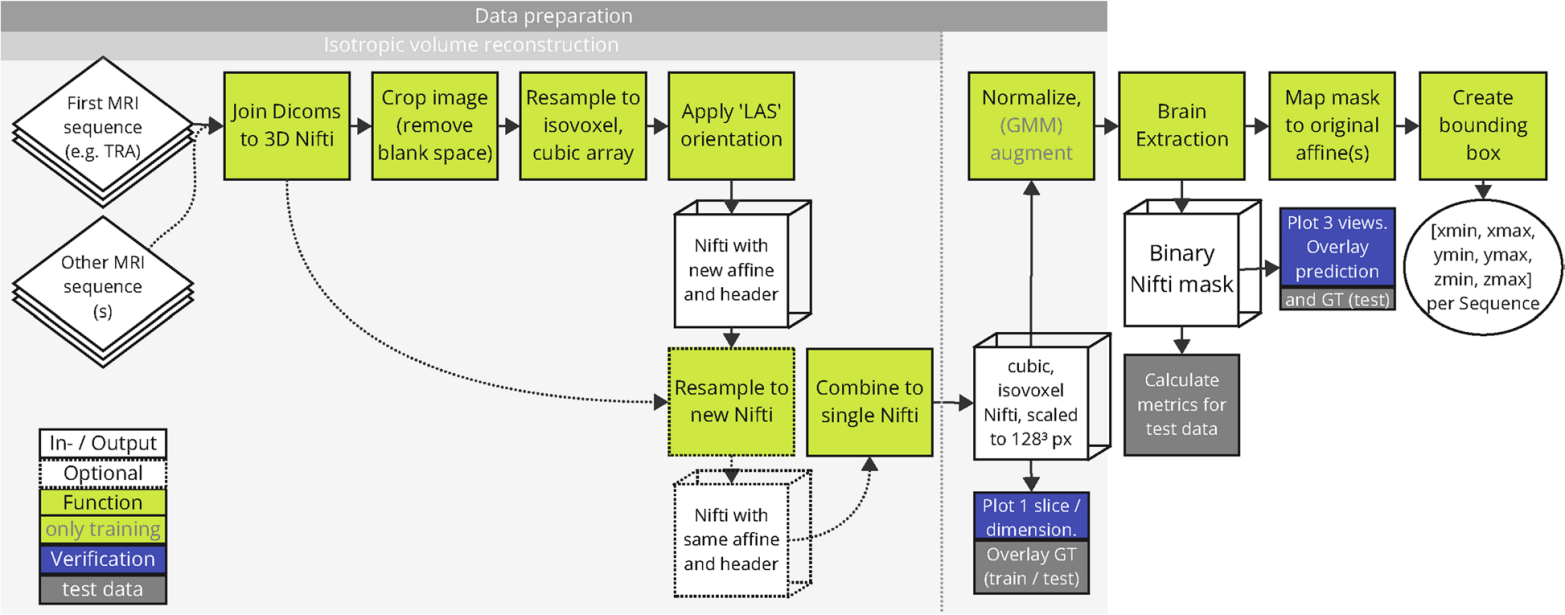
Flow chart of the transformation of DICOM files to NIfTI mask file

The preprocessing workflow for our brain extraction model composed of cropping, resampling, and file format conversion (Fig. 1). We implemented the algorithms in Python using the modules *dicom2nifti* [16], *nibabel* [17], and *nilearn* [18] for registration of the images to the target format.

#### Isotropic volume reconstruction

According to Avesta et al., brain extraction models work best for 3D volume data [19]. To make use of the resolution of each acquisitional plane, we combined the image stacks into one single 3D image and mapped them back to their original affine matrices post prediction. It was also more time effective, to perform resampling, reorientation, and prediction on the combined image, rather than separately for each subsectional plane. The algorithm worked for one up to three scan orientations of DICOM image stacks. The preprocessing started by converting the DICOM slices into a Neuroimaging Informatics Technology Initiative (NIfTI)-coded file. Then, we cropped the TRA images removing any black space and resampled them to an isovoxel, standardized ’LAS’ (Left-Anterior-Superior) oriented, cubic image with $128^3$ pixels. This standardization of orientation addresses the variability in acquisition protocols across different scanner vendors and institutions. The voxel sizes were normalised to create cubic voxels while preserving the original anatomical proportions, accommodating variations in slice thickness and in-plane resolution across different scanners. If the data file did not provide a TRA sequence, we substituted another acquisition plane for the volume reconstruction. After the initial registration to the target format, we resampled the other orientations to the same affine and combined all available 3D volumes by choosing the maximum intensity value at each voxel position across all orientations, which preserves the highest signal from each acquisition plane. Our pipeline includes an alignment analysis, that detects poorly aligned sequences, that have a center offset greater than 10 mm or volume overlap less than $30\%$, and reprocesses them separately to ensure consistent spatial relationships across heterogeneous data. Variable blank space around the anatomy was handled through automatic cropping, which focuses computational resources on relevant anatomy while maintaining consistent spatial context.

This approach ensured that despite differences in field of view and patient positioning across institutions, all images maintained consistent spatial properties for subsequent analysis. We stored the original affine of the images to enable realignment to their original slices, size, position, and orientation.

#### Image augmentation

To increase the number of training samples, we augmented the 3D images. We applied commonly used augmentation methods in medical imaging including flipping, rotation, zoom. For intensity-based augmentation, we employed Gaussian mixture model (GMM) to model and modify the intensity distributions. For each augmentation, we randomly selected between 1 and 3 Gaussian components. The component means were perturbed with random values drawn from a uniform distribution in the range [0.01, 3.0], while standard deviations were altered using values from [0.01, 0.1]. These parameter ranges were empirically determined to create realistic variations in tissue contrast while preserving anatomical structures.

To keep the data uniform and close to the real images, we further augmented the samples applying the following techniques:

1. flipping in the transversal plane, i.e. reversing the orientation from left to right,
2. head rotation along the right–left (RL) axis, corresponding to anterior-posterior movement
3. zooming in on images containing a significant proportion of non-brain tissues
4. zooming out on all other images.

We controlled the scale of the zoom manually for each 3D image, since some MRI sequences of our dataset were too tightly focused on the brain region and therefore zooming in would undesirably remove parts of the region of interest (ROI). For all training images, we also adjusted the rotations manually to cope with undesired shifts of the brain region out of the image centre. The manual control of rotation and zoom factors was only applied during the creation of training data augmentations to ensure appropriate variability that reflects real-world clinical scenarios. Our operational pipeline for test data ran without any manual intervention.

#### Reduction of scanner bias

In addition to standardising data shapes, several methods were applied to reduce the scanner bias. For benchmarking, we trained the model on a subset of the insitution A and tested on the other instances of the same institution and the datasets of the three remaining institutions (B-D). Differences in the prediction scores between the known institution and the unseen institutions, mainly caused by the scanner bias, were reduced by image preprocessing and augmentation techniques. According to Meyer et al. [20], we combined N4 bias field correction [21, 22], z-score normalisation per training case, and GMMs [20] for augmentation. The N4 bias field correction was applied with a shrink factor of 10 and binary mask threshold at the 60th percentile of image intensity. This correction step was performed first, to address the intensity non-uniformities introduced by different scanner hardware, before z-score normalisation was used to standardise intensity distributions across acquisitions with varying contrast characteristics. The GMM-based augmentation, was subsequently performed on the training dataset. The test data was bias-corrected and z-score normalised for best results.

### Further modalities

**Fig. 2:**
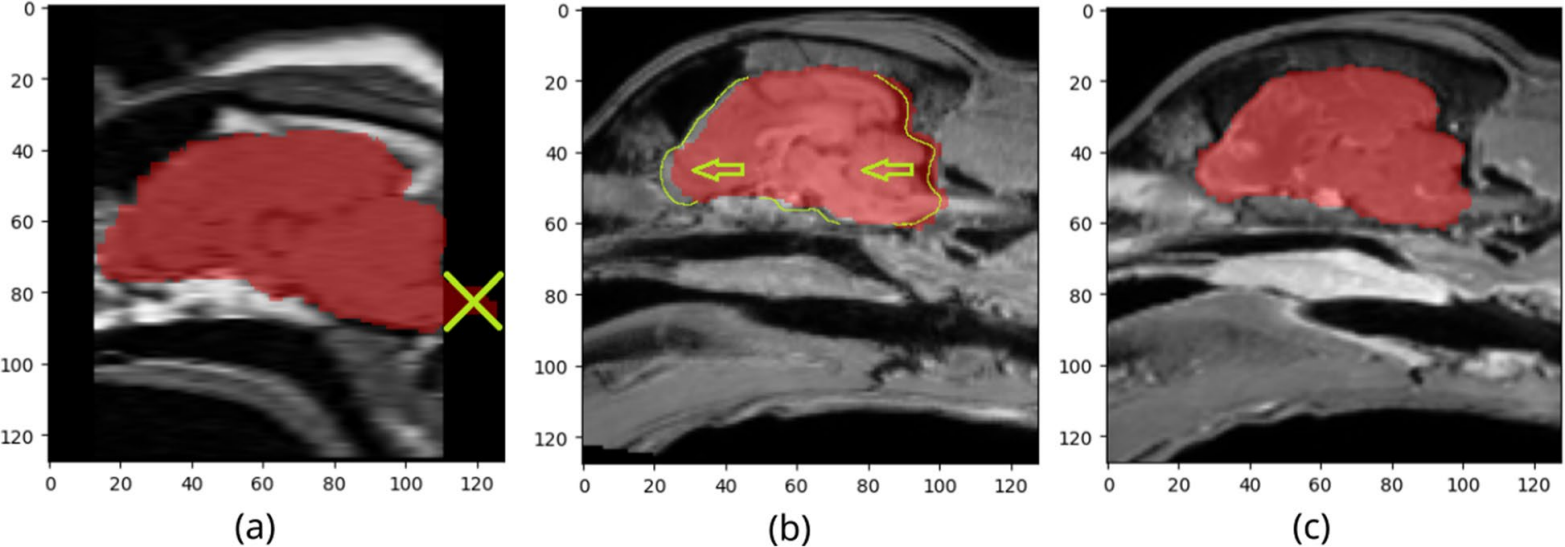
Incorrect masks after mapping images from other modalities to match masks created for T1ce images. Mask (**a**) crosses over image bounds. It should end with the image boundaries - area crossed out in green. Mask (**b**) shows an image from a shifted position. It should allocate a few pixels to the left. **c** shows the mask position of (**b**) on the original image

To test the brain extraction algorithm on T1, T2, and T2-FLAIR sequences, we mapped the corresponding images to the same affine as the T1ce image of the same subject. Due to repositioning of the dogs between the recordings, some images did not map properly (Fig. 2b). If the new image modality didn’t include the entire image volume, we cropped the mask to the new image boundaries, to avoid faults in the metric calculation (Fig. 2a). We tested only on images with matching ground truth masks. As we only mapped the images of these modalities to match the masks of their T1ce counterparts, the ground truth mask might differ from what an expert would have labelled manually.

### Model training and testing

We deployed the preprocessing pipeline and the neural network on a i9 10920x CPU and a graphic card (NVIDIA GeForce RTX 3080 Ti GPU with 12GB of VRAM). We trained the 3D nnU-Net [15] for 100 epochs preventing overfitting without significantly affecting the prediction since the validation curve already converged at this stage of training. As optimiser, we used stochastic gradient descent (SGD) with momentum as it generalises better than adaptive optimisation methods, such as adaptive moment estimation (ADAM) [23]. Apart from these adjustments we used the standard trainer with an initial learning rate of 0.01, default settings, and without pre-trained weights.

We tested the algorithm and models on data that we did not use during training. Regarding the multiple strata of the target domain, we applied different breeds, head sizes, canine cranial conformations, scanner sites, MRI modalities (T1, T1ce, T2, and T2-FLAIR), and numbers of acquisition planes. We validated the results by comparing the predicted brain segmentations to the manually created ground truth through the metrices dice coefficient (Dice), intersection over union (IoU), which is also referred to as Jaccard index, sensitivity, and specificity. Then, we measured the impact of brain extraction as a preprocessing step to a classification model with gain of accuracy.

### Single-site model

To show the models’ dependence on the scanner, it was only trained on images from institute A and tested on the validation set and data from the three other sites (B to D), with and without GMM augmentation.

### Multi-scanner model

For the final multi-scanner model, we included three scanner sites (A to C) during training. After augmentation, each split of the 5-fold training contained 1356 training and 339 validation 3D images. To avoid memorizing the individual masks instead of learning a general brain extraction, we applied a leaving one dog out strategy, i.e., the dogs’ data is separated into either the training or the test set. The combinations of training and validation data showed different tendencies for overfitting. As the final model, we chose the best-performing fold and did not combine all models, as the increase in performance was not substantial but the processing time increased remarkably. We post-processed the prediction results to contain only the largest connected region of the mask and exclude artefacts far from the brain center. We performed the final testing of the model on previously unused, non-augmented images from institute D. In addition to the training modality T1ce, we tested the algorithm on three other MRI modalities, namely T1, T2, and T2-FLAIR.

### Brain extraction as preprocessing step

We applied brain extraction as preprocessing for a convolutional neural network (CNN) classifying slices of dog T1ce and T1 MRI images into normal and abnormal. We calculated a bounding box out of the brain extraction masks, cropped the images to include only the ROI of the brain tissue and performed histogram-based normalisation. Brain extraction was necessary to obtain robust normalisation results. We trained the CNN on the original data and on the cropped, normalised images. With this we aimed to reduce the complexity of the model and to enable a focus on solely detecting diseases, rather than distinguishing non-brain tissues.

## Results

Our brain extraction algorithm resulted in a single-scanner and a multi-scanner model. We followed the test procedure described in the previous sections and compared to the reference brain extraction method for dog MRIs.

### Model performance

#### Single-site model

**Fig. 3:**
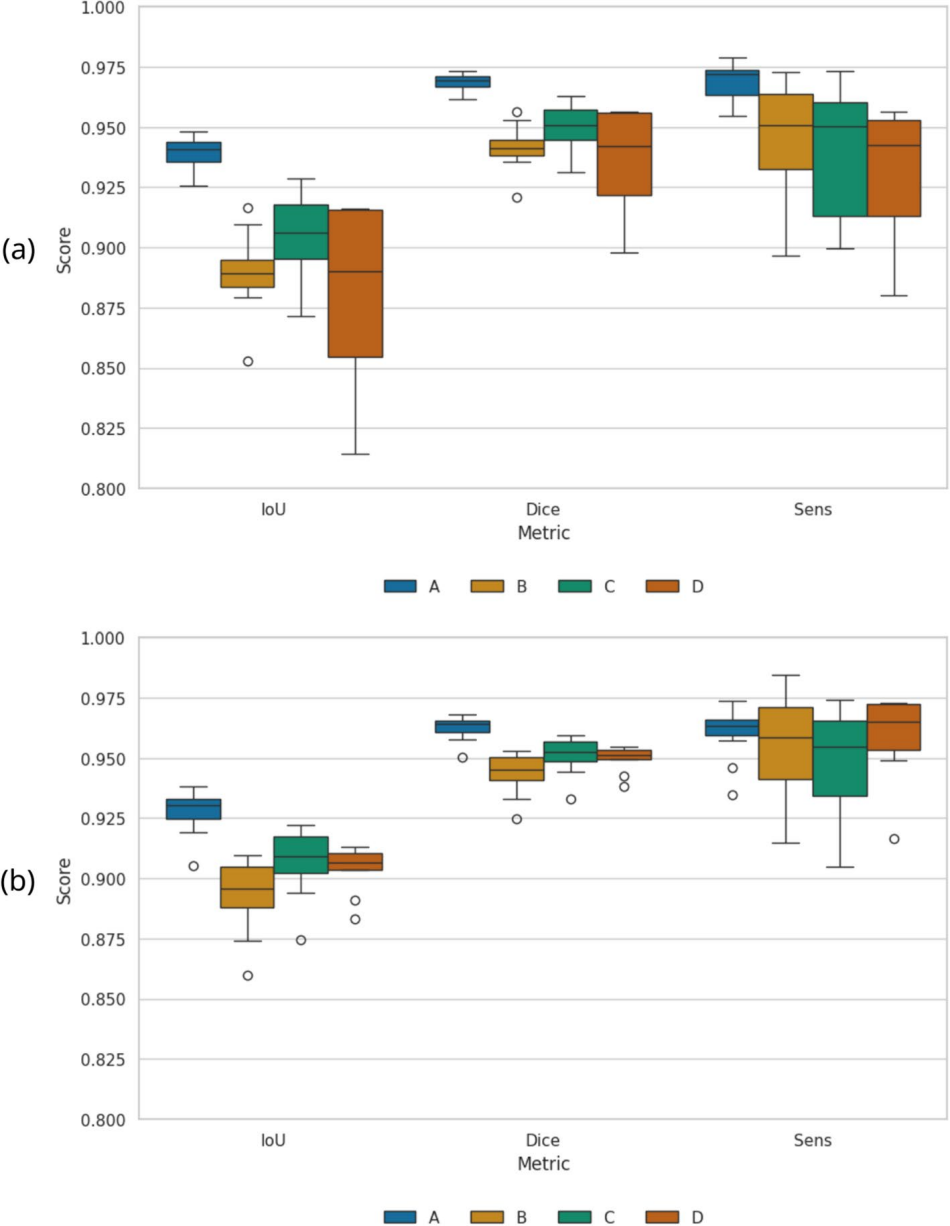
Scanner bias on single site model. Trained on images from institute A, tested on sites B-D. Box plots show the quartiles of the dataset, with a line indicating the median. Whiskers extend to show the rest of the distribution, excluding outliers (circles). **a** Data augmentation only, per institute. **b** Additional GMM augmentation, per institute

GMM augmentation significantly reduced the variability in predictions of unseen scanners and only slightly reduced the performance on the validation set of the known scanner (Fig. 3). Without GMM augmentation, the model trained only on scanner A achieved a mean IoU of $0.88\pm 0.03$ on scanners A to C, but only $0.31\pm 0.36$ on one of the new scanners (D). As there was no statistically relevant difference between the known scanners from A and the unknown scanners from B and C, we decided to keep the images of scanner D as a testing dataset, as it best tracks the reduction of the scanner bias. With GMM augmentation, the performance on the problematic scanner D improved dramatically to $0.89\pm 0.04$ IoU, demonstrating the critical role of our augmentation approach in reducing scanner bias. The overall performance also slightly increased to $0.90\pm 0.03$ IoU.

#### Multi-scanner model

**Fig. 4:**
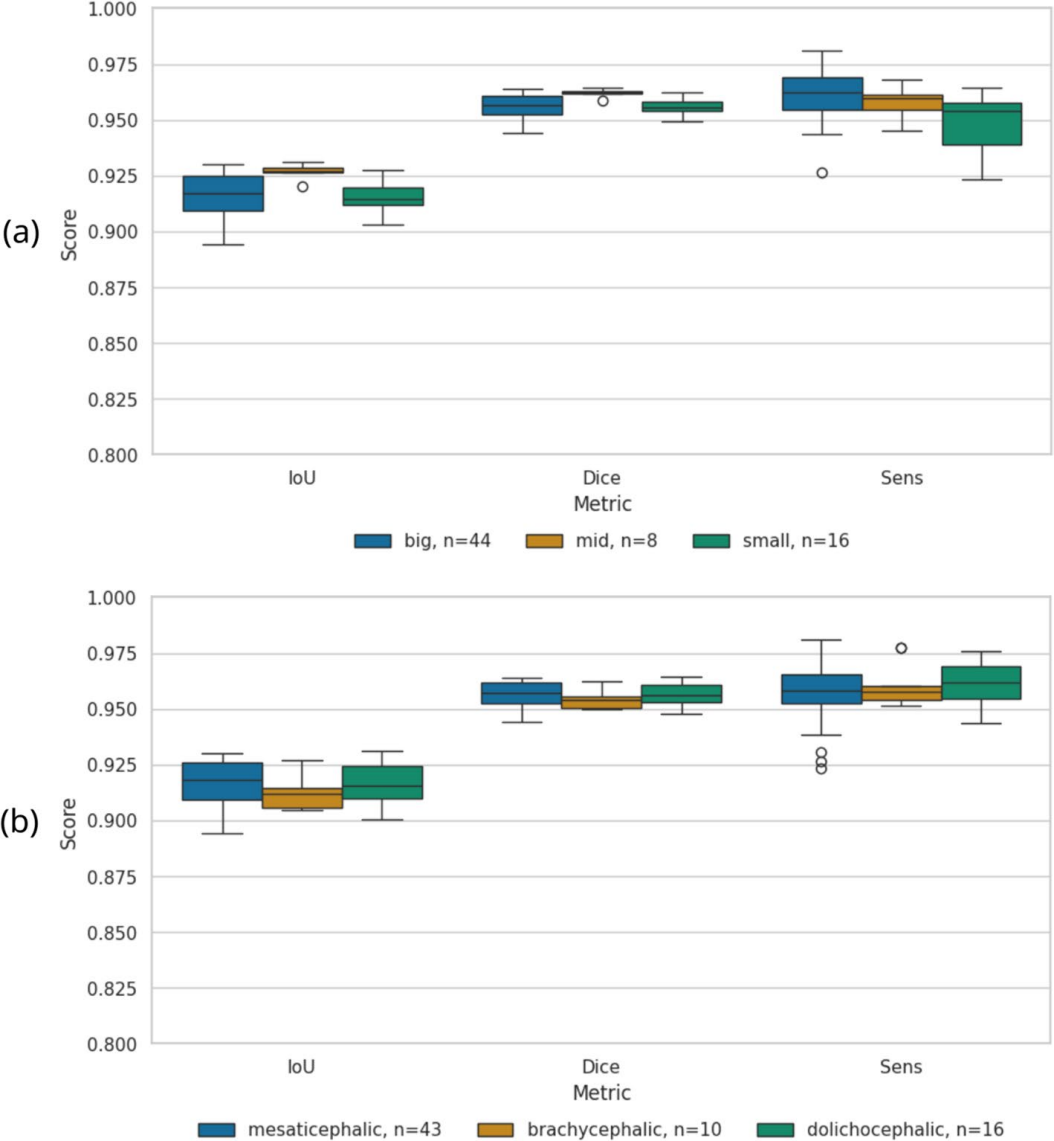
Boxplots of prediction results over influencing factors head size and cranial conformation. We make predictions on same dogs as used in training, but without any augmentations. **a** Comparison over dog size. The dogs were divided into three groups of sizes by weight: small (< 5.5kg), medium (5.5kg to 25 kg) and big (25kg). **b** Comparison over cranial conformation

Since the model was trained on this modality, we expected best prediction results on T1ce images. Indeed, the segmentations were only slightly less accurate for the new scanner D (Table 3). We report the results as average values and include the standard deviation over the test set. The model also generalised well over all head sizes and cranial conformations (Fig. 4).

**Table 3:** Multi-scanner results on T1ce images

Dataset	IoU	Dice	Sens	Spec
A-C	0.91±0.01	0.95±0.01	0.95±0.01	0.998±0.001
D	0.90±0.01	0.95±0.00	0.96±0.01	0.998±0.001
combined	0.90±0.01	0.95±0.01	0.96±0.01	0.998±0.001

Results are divided into unseen scanner D, validation data of known scanners A-C, and the combination of both

**Fig. 5:**
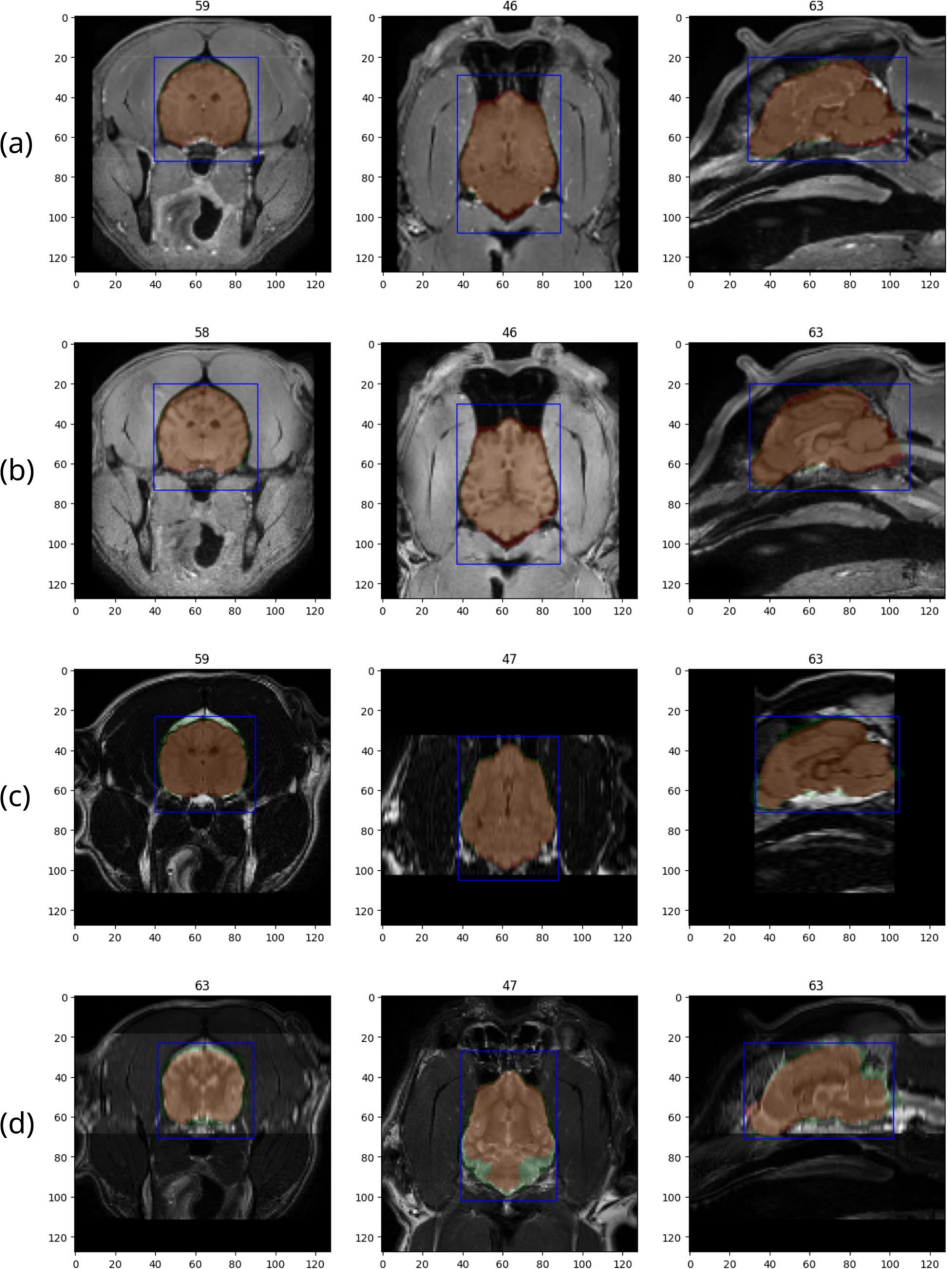
Example predictions on scanner site D, for T1ce (**a**), T1 (**b**), T2-FLAIR (**c**), and T2 (**d**) images. Subject is a mesaticephalic Labrador with no apparent pathologies. The volume is sliced in the middle of the derived bounding box (blue) for each axis. Ground truth masks (green) are superimposed with predictions (red). Intersections are shown in orange. The prediction results are (**a**) T1ce, IoU 0.89, dice 0.94, sensitivity 0.94, specificity 0.9979; (**b**) T1, IoU 0.87, dice 0.93, sensitivity 0.96, specificity 0.9968; (**c**) T2-FLAIR, IoU 0.82, dice 0.9, sensitivity 0.83, specificity 0.9994; (**d**) T2, IoU 0.72, dice 0.84, sensitivity 0.73, specificity 0.9997

Despite training only on T1ce images, the augmentation introduced sufficient variations of intensities and contrast that the prediction adapted well to these modalities (Fig. 5). While slight variations of the ground truth mask from the underlying image impacted the resulting scores, manual inspection still showed very accurate predictions. The performance also remained consistent for only a single acquisition plane, supporting the model’s capability across various acquisition conditions (Fig. 5c).

Preprocessing with bias correction and z-score normalisation resulted in an average IoU improvement of $2\%$ across all modalities, with more substantial improvements of $6\%$ for predictions on T2 images. The normalisation techniques also led to more consistent results with lower standard deviations across all metrics.

**Fig. 6:**
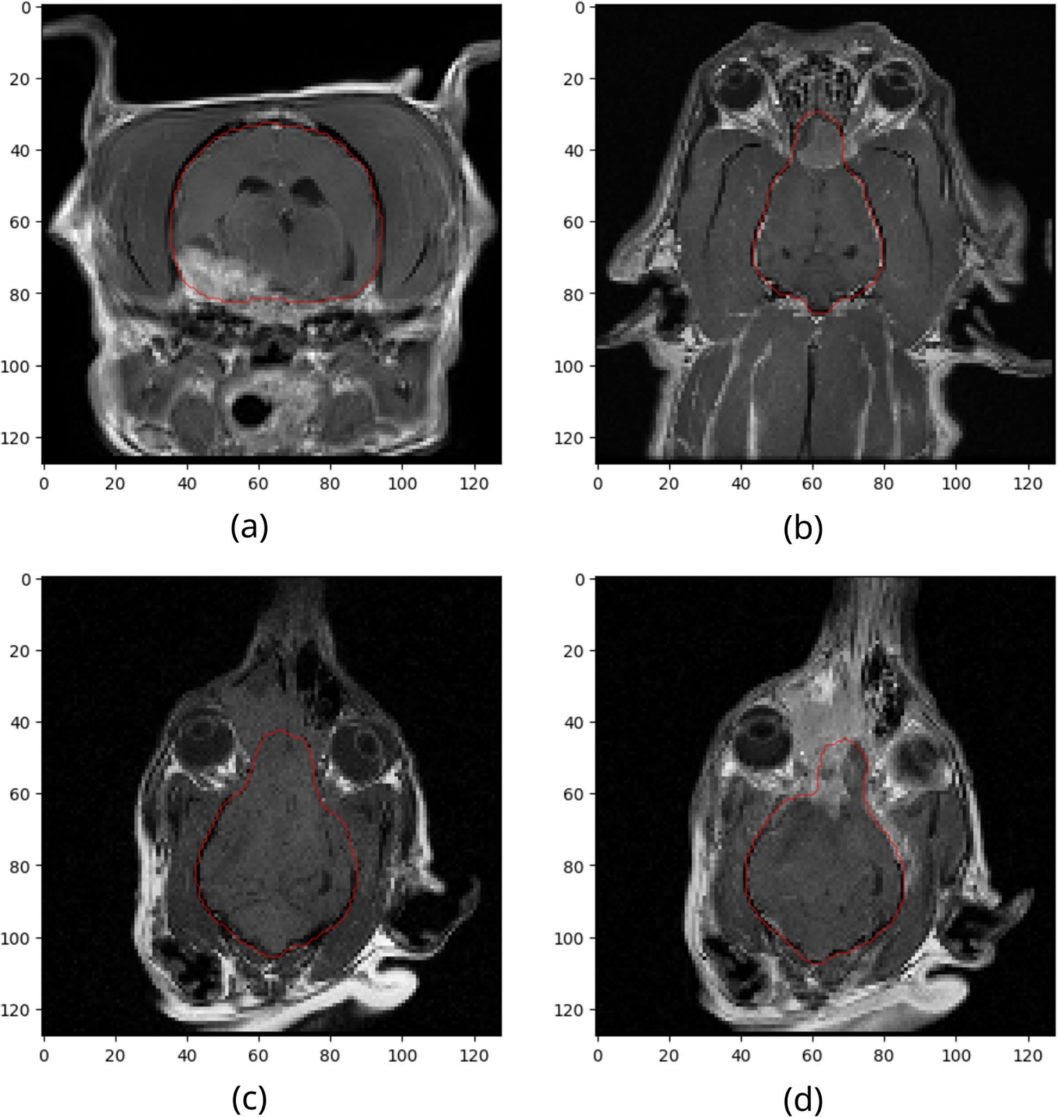
Transversal (**a**) and dorsal (**b** - **d**) T1 sequences of canine brains with pathological conditions. The red line represents the external border of the brain after automatic skull stripping. **a** transversal T1ce imaging of a canine brain at the level of the thalamus. There is an extraaxial, contrast enhancing mass at the right piriform lobe. **b** dorsal T1ce imaging of a dog with an extraaxial mass in the left olfactory bulb with marked contrast enhancement. **c** dorsal pre-contrast (T1) imaging of a dog with an extraaxial mass in the right olfactory bulb, invading the frontal sinus. **d** dorsal post-contrast (T1ce) imaging of a dog with an extraaxial mass in the right olfactory bulb with marked contrast enhancement invading the frontal sinus

Critical areas, often slightly conflicting with the ground truth masks were the transition from brain to cerebrospinal fluid (CSF) (6 out of 10), and the transition of the brainstem to the spinal cord (3 out of 10). Still, the predictions on the trained modality T1ce included all relevant regions, with no tendency for over- or underestimation. For the untrained modality T1, the model overestimated in 4 of 10 cases (Fig. 5b). We observed some cases of underestimated brain segmentations in the modalities T2 and T2-FLAIR (Fig. 5d). Challenging features of pathologies in the thalamus or the rostral cerebrum in doliocephalic canines did not negatively affect the performance of the brain segmentation, as visual analysis of superimposed segmentation masks on MRIs confirmed the high accuracy of the model, even in the presence of severe pathologies (Fig. 6).

### Computational resources

Measured on the same NVIDIA GPU from training, the model used about $1.9 \textrm{GB}$ VRAM for prediction with and without overhead. The 3D alignment and further preprocessing of the volumes took on average $4 \textrm{s}$, depending on the original image resolution and amount of DICOM files to process. The brain extraction itself took approximately $1.5 \textrm{s}$, including post-processing. Performing the inference on the CPU took around $25 \textrm{s}$. Mapping the mask back to its original space added less than a second to the computation time. In total, this added up to an average time per brain extraction of $6 \textrm{s}$ or $30 \textrm{s}$ on GPU and CPU, respectively. The duration stayed almost the same for multiple sequences acquired in the same scan.

### Model benchmarking

We compared our algorithm with VIBE, the only other existing method for canine brain extraction [7]. VIBE is designed and tested for clinically normal dogs only. Our nnU-Net model outperformed VIBE by $3\% \mathrm {~IoU}$, $2\% \mathrm {~Dice}$, $1\% \mathrm {~sensitivity}$, and $0.3\% \mathrm {~specificity}$ when comparing the target modalities T1 BRAVO and T1ce (Table 4). It also scored higher on the T2-FLAIR sequences ($9\% \mathrm {~IoU}$, and $5\% \mathrm {~Dice}$). However, in terms of sensitivity, it was inferior to the atlas-based method for T2 and T2-FLAIR sequences. The variability in prediction results was greater for modalities not seen in training.

**Table 4:** Multi-scanner results, and scores of atlas-based canine brain extraction *VIBE*

Dataset	IoU	Dice	Sens	Spec	Duration
VIBE T1 BRAVO	0.87 ± 0.03	0.93 ± 0.02	0.95 ± 0.02	0.995 ± 0.003	$90\textrm{s}..110\textrm{s}$
VIBE T2 FLAIR	0.80 ± 0.03	0.89 ± 0.03	0.93 ± 0.05	0.993 ± 0.004	$15\textrm{s}$
T1ce	0.90±0.01	0.95±0.01	0.96±0.01	0.998±0.001	6 s (GPU)
T1	0.88±0.03	0.94±0.01	0.96±0.01	0.997±0.002	30 s (CPU)
T2	0.86±0.04	0.92±0.02	0.92±0.05	0.998±0.001	
T2-FLAIR	0.89±0.02	0.94±0.01	0.92±0.03	0.999±0.000	

Results of the mean and standard deviation results of the dice similarity coefficient and IoU for four different modalities (T1, T1ce, T2, and T2-FLAIR) are from combined scanner sites A-D. Durations were measured on a MacBook Pro (VIBE) and an NVIDIA GeForce RTX 3080 Ti GPU with 12GB VRAM and i9-10920X CPU

The computation times differed with the original image sizes. Due to the downsampling within the preprocessing pipeline, the nn-Unet performed predictions faster on large-size images, whereas VIBE may be faster on low resolution images, when compared on similar working stations.

### Brain extraction as preprocessing step

As a preprocessing step of a CNN classifying slices of dog T1ce and T1 MRI images into normal and abnormal, we applied brain extraction, cropped the images to the ROI and applied normalisation to the images. Compared to using the original images, this improved the classification accuracy, by $10\%$ (Table 5).

**Table 5:** Scores of a classification model with and without cropping to the ROI and normalisation

	Normal		Abnormal	
	Full image	Cropped to ROI	Full image	Cropped to ROI
Precision	0.93	0.93 (+0%)	0.40	0.50 (+10%)
Recall	0.59	0.73 (+14%)	0.87	0.84 (−3%)
F1-score	0.72	0.82 (+10%)	0.55	0.62 (+7%)
Accuracy	0.66 (full image) $\rightarrow$ 0.76 (cropped to ROI) (+10%)			

## Discussion

This study presents a deep learning (DL) method for automatic brain extraction, applicable for MRI scans with pathological conditions in dogs. The algorithm generates a 3D image in isotropic voxel representation from DICOM sequences. The brain extraction performs on the image volume and the resulting masks reallocate to the original sequence(s). The approach is designed for strongly pathological T1ce images, but also works well for dogs with healthy brain or subtle structural changes as well as images from other modalities, without additional training or tuning. Despite reducing the scanner bias by augmentation, prediction results are more precise for images of trained scanners and modalities.

The generalisation capability of the multi-scanner model proved robustness across varying head sizes and cranial conformations, further supporting its wide applicability. Slight irregularities at brain-to-CSF or brainstem-spinal-cord transitions might be caused by inconsistent labelling or undesired effects during the volume reconstruction from 2D slices. These findings suggest that including various modalities into training could improve generalisation across MRI acquisition types. To further improve the model, one could explore different algorithms for image resampling, to speed up the alignment to the target image format. To enhance the level of detail in the segmented mask for higher-resolution images, a subsequent network could be implemented, as suggested by Svanera et al. [24].

Classification of abnormalities in canine MRIs image slices improve by performing brain extraction as a preprocessing step. We expect that brain extraction has even more impact when faced with the more complex task of classifying different diseases. Moreover, brain extraction supports multiple other applications in the veterinary field, such as segmentation of lesions.

Our results further show that the alignment of different modalities can be done by mapping the segmented masks. This in turn enables robust multi-modal classifications and likely increase classification results, as some symptoms are only visible on varying MRI imaging types.

While our final test set was limited to 10 dogs with ground truth masks, we additionally validated the algorithm through manual review of approximately 50 further cases, with consistently promising results. Although our approach has reached a routine-usable stage, further validation, particularly across diverse scanner sites and breeds, is essential to ensure generalisability. Critical transitions, such as brain to CSF and brainstem to spinal cord, remain areas for refinement due to inconsistencies in available ground truth annotations.

Future work should focus on further improving the model’s generalisability by including a broader range of modalities in training and exploring more advanced algorithms for image resampling and segmentation. Overall, this study not only lays a strong foundation for automated brain extraction in veterinary applications but also opens avenues for more complex diagnostic and multi-modal classification tasks.

## Conclusion

Our research presents a robust and highly adaptable deep learning method for automatic brain extraction from canine MRI scans, demonstrating significant performance improvements over existing methods, particularly for T1ce images. The algorithm shows promising generalisation across different MRI modalities, although we noted some variability in predictions for non-trained modalities. Our approach is computationally efficient, as the entire preprocessing and brain extraction process takes an average of $6\textrm{s}$ per scan. Using our brain extraction as preprocessing enhances the accuracy of subsequent CNN-based classification by $3\%$.

## Data Availability

The datasets generated and analyzed during the current study are not publicly available due to patient confidentiality and restrictions imposed by German data protection regulations (DSGVO). However, data may be made available upon reasonable request and subject to appropriate data protection agreements. Requests should be directed to the corresponding author.

## Appendix

### Manual labelling

The set of canine brain MRIs is mapped in consensus labelling by experts from the University of Veterinary Medicine Hanover (TiHo). The labelling is done in a slice-by-slice manner on all scan levels TRA, DOR, and SAG, with help of the annotation tool [25]. We defined the boundaries for each canine brain segment to include the maximum extent of the brain in each of the planes.

In particular, masks in the midsagital plane must include the brainstem up to the level of the foramen magnum to the most rostral part of the olfactory bulb, and from the cortex of the parietal area to the ventral part of the brainstem and the pons as well as the olfactory bulb including the pituitary gland between both.

In context of creating the brain masks, brain tumors and other diseases belong to the brain tissue.
